# Cucurbitacin B Inhibits the Proliferation of WPMY-1 Cells and HPRF Cells via the p53/MDM2 Axis

**DOI:** 10.3390/ijms25179333

**Published:** 2024-08-28

**Authors:** Yangtao Jin, Ping Zhou, Sisi Huang, Congcong Shao, Dongyan Huang, Xin Su, Rongfu Yang, Juan Jiang, Jianhui Wu

**Affiliations:** 1 NHC Key Lab of Reproduction Regulation, Shanghai Engineering Research Center of Reproductive Health Drug and Devices, Shanghai Institute for Biomedical and Pharmaceutical Technologies, Pharmacy School, Fudan University, Shanghai 200237, China; jyt_sphu@163.com (Y.J.); zhouping19a@163.com (P.Z.); sisihuang666@163.com (S.H.); shaocongcongscc@163.com (C.S.); hdy043@163.com (D.H.); suxiaoxin1982@163.com (X.S.); yangrongfu82@163.com (R.Y.); jiangjuan06@126.com (J.J.); 2 Department of Pharmacology & Toxicology, Shanghai Institute for Biomedical and Pharmaceutical Technologies, Shanghai 200032, China

**Keywords:** triterpenoid compound, cucurbitacin B, prostatic hyperplasia, p53 signaling pathway

## Abstract

Modern research has shown that Cucurbitacin B (Cu B) possesses various biological activities such as liver protection, anti-inflammatory, and anti-tumor effects. However, the majority of research has primarily concentrated on its hepatoprotective effects, with limited attention devoted to exploring its potential impact on the prostate. Our research indicates that Cu B effectively inhibits the proliferation of human prostate stromal cells (WPMY-1) and fibroblasts (HPRF), while triggering apoptosis in prostate cells. When treated with 100 nM Cu B, the apoptosis rates of WPMY-1 and HPRF cells reached 51.73 ± 5.38% and 26.83 ± 0.40%, respectively. In addition, the cell cycle assay showed that Cu B had a G2/M phase cycle arrest effect on WPMY-1 cells. Based on RNA-sequencing analysis, Cu B might inhibit prostate cell proliferation via the p53 signaling pathway. Subsequently, the related gene and protein expression levels were measured using quantitative real-time PCR (RT-qPCR), immunocytochemistry (ICC), and enzyme-linked immunosorbent assays (ELISA). Our results mirrored the regulation of tumor protein p53 (TP53), mouse double minute-2 (MDM2), cyclin D1 (CCND1), and thrombospondin 1 (THBS1) in Cu B-induced prostate cell apoptosis. Altogether, Cu B may inhibit prostate cell proliferation and correlate to the modulation of the p53/MDM2 signaling cascade.

## 1. Introduction

Benign prostatic hyperplasia (BPH) is the most common cause of lower urinary tract symptoms (LUTS) in men and is characterized by epithelial and stromal cell proliferation. With the acceleration of global aging, the incidence rate of prostatic hyperplasia has increased. An epidemiological study suggested that global BPH patients exceeded 210 million in 2010 [1]. The absolute number of incidences increased considerably, from 5.48 million in 1990 to 11.26 million in 2019 [2]. The incidence of BPH increases with age, and up to 50% of men over 50 years old and 80% of men over 80 years old will experience LUTS caused by BPH [3]. Thus, concomitant with the extension of the average expectant lifespan, benign prostatic hyperplasia will become a global public health issue.

Nowadays, for the majority of BPH patients with mild symptoms, pharmacotherapy would be the preferred route of clinical treatment. The commonly used chemical drugs for the treatment of BPH are divided into two categories, namely 5-alpha reductase inhibitors and alpha-adrenergic receptor antagonists. The growth and development of the prostate depend on the stimulation of androgens. In the stroma and basal cells of the prostate, testosterone is converted into dihydrotestosterone with stronger androgenic effects by 5-alpha reductase, thus stimulating excessive proliferation of prostate cells [4]. Clinically, 5-alpha reductase inhibitors such as finasteride, dutasteride, and epristeride are commonly used to inhibit the conversion of testosterone to dihydrotestosterone, thereby improving BPH. In addition, G protein-coupled adrenergic receptors can activate prostate and vascular smooth muscle contraction, promote proliferation response, and regulate cytoskeletal proteins in prostate smooth muscle cells [5]. Alpha-1 blockers, including terazosin and doxazosin, can alter the tension around the prostatic urethra by targeting alpha-1 adrenergic receptors, thereby relieving obstructive symptoms caused by BPH. 5-alpha reductase inhibitors and alpha-adrenergic receptor antagonists can reduce prostate volume and mitigate BPH-correlated LUTS symptoms to a certain extent. Nonetheless, 5-alpha reductase inhibitors and alpha-adrenergic receptor antagonists showed various side effects over time, including postural hypotension, headache, and ejaculatory dysfunction [6]. Accordingly, it is of paramount importance to develop efficient and low-toxicity therapeutic agents for benign prostatic hyperplasia.

Natural products include components or metabolites from animals, plants, insects, marine organisms, and microorganisms that have significant medicinal and economic values [7]. Natural products exhibit excellent pharmacological properties, targeting multiple therapeutic targets efficiently while showing minimal side effects [8]. Therefore, the utilization of natural products is anticipated in the therapy of prostatic hyperplasia diseases. Triterpenoids are an important category of natural products, consisting of six isoprene units with various modifications to their carbon skeletons and complex and diverse structures [9]. Overall, triterpenoid compounds exhibit effective antifungal, antibacterial, and hepatoprotective activities, and more importantly, they possess extensive anticancer activities [10]. Ginsenoside Rh2 suppresses glioma proliferation through modulation of the miR-128/E2F3a signaling pathway, resulting in enhanced cytotoxicity, apoptosis, and caspase 3 activation [11]. Ursolic acid can engage in lung cancer prevention efforts by mitigating the DNA damage protection mediated by vaccinia-related kinase 1 [12], suppressing the epithelial–mesenchymal transition through down-regulation of astrocyte-elevated gene-1 [13], and activating the stress-activated kinase/c-Jun N-terminal kinase (SAPK/JNK) signaling pathway [14]. Clearly, natural products, including triterpenoids, have broad potential for treating various types of cancer, and it is necessary to explore and study them comprehensively. This study screened and identified triterpenoid compounds with anti-proliferative activity against prostate cells from a purchased natural product compound library and found that cucurbitacin B has good activity.

**Figure 1:**
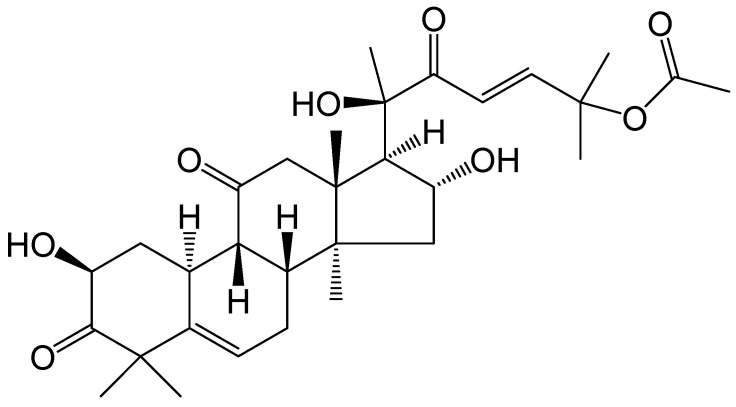
The chemical structure of cucurbitacin B.

Cucurbitacin B (Cu B) is a tetracyclic triterpenoid compound isolated from cucurbitaceae and other plants with a wide range of pharmacological activities (Figure 1). Cu B, the most abundant and active triterpenoid in the cucurbitacin family, has been confirmed by modern pharmacological studies to exhibit significant therapeutic effects on inflammatory diseases, neurodegenerative diseases, diabetes, and cancer, among others [15]. However, the exact function and potential molecular mechanisms of Cu B in prostate cells have not received sufficient attention. Recent studies have found that Cu B can exert anticancer effects by inducing apoptosis and cell cycle arrest in human prostate cancer PC3 cells through down-regulation of the JAK/STAT signaling pathway [16], as well as inhibiting prostate cancer growth by inactivating ATP-citrate lyase [17]. In our previous research, we found that Cu B could exert antiproliferative and apoptotic effects on BPH-1 cells through the p53/MDM2 axis [18]. In this research, the impact of Cu B on the proliferation and apoptosis of two distinct cell lines, namely the immortalized human normal prostate stromal cell line (WPMY-1) and human prostate fibroblasts (HPRF), was quantitatively assessed through the utilization of the Counting Kit-8 (CCK-8) assay and flow cytometry techniques. Employing RNA sequencing technology, we delved into the molecular mechanisms underlying the inhibitory effect of Cu B on WPMY-1 cell proliferation. Specifically, we monitored the expressions of tumor protein p53 (*TP53*), mouse double minute-2 (*MDM2*), thrombospondin 1 (*THBS1*), and cyclin D1 (*CCND1*) genes and proteins using real-time quantitative polymerase chain reaction (RT-qPCR), enzyme-linked immunosorbent assays (ELISA), and immunocytochemistry. Our research revealed that Cu B served as a proapoptotic agent, effectively suppressing prostate cell proliferation. This suppression was achieved by augmenting the expressions of TP53 and THBS1 while concurrently diminishing the expressions of MDM2 and CCND1. Conclusively, our study revealed the antiproliferative effect of Cu B on prostate cells, which laid the groundwork for future research related to Cu B as a therapeutic agent for benign prostatic hyperplasia.

## 2. Results

### 2.1. Cu B Exhibited Antiproliferative Effects on WPMY-1 and HPRF Cells

**Figure 2:**
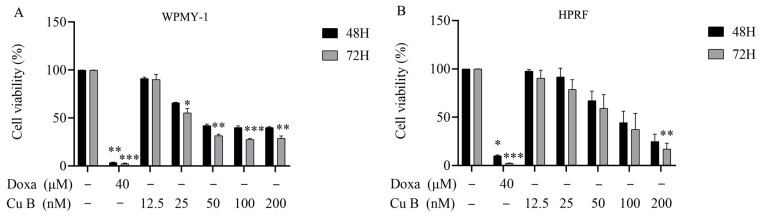
Effect of Cu B on the cell viability of WPMY-1 (**A**) and HPRF (**B**). Cu B and Doxa inhibited the proliferation of WPMY-1 and HPRF cells based on CCK-8 results. Prostate cells were treated with vehicle (0.1% DMSO), doxazosin (40 μM), or Cu B (12.5 nM, 25 nM, 50 nM, 100 nM, 200 nM) for 48 h and 72 h. The positive control drug doxazosin 40 μM significantly reduced the survival rate of both cells compared with the solvent control group. After 72 h of treatment with Cu B at medium to high concentrations (25 nM–200 nM), the survival rate of WPMY-1 cells was significantly reduced compared with the solvent control group. Only after 72 h of treatment with the highest concentration (200 nM) of Cu B was the survival rate of HPRF cells significantly reduced compared with the solvent control group. The data are presented as mean ± standard deviation (SD) of three independent experiments, analyzed using one-way analysis of variance (ANOVA), followed by least significant difference post-hoc test or Dunnett’s post-hoc test. * *p* < 0.05, ** *p* < 0.01, *** *p* < 0.001, compared with the control group. Cu B, cucurbitacin B; Doxa, doxazosin; CCK-8, counting kit-8; WPMY-1, human normal prostate stromal immortalized cell line; HPRF, human prostate fibroblasts; DMSO, dimethyl sulfoxide.

**Figure 3:**
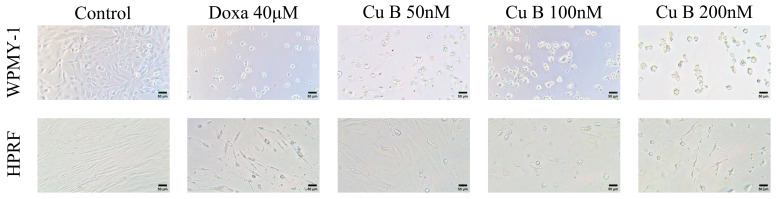
Effect of Cu B (50 nM–200 nM) and doxazosin (40 μM) on the cellular morphology of WPMY-1 and HPRF. Compared with the control group, Cu B treatment induced distinct morphological alterations, manifesting with cell shrinkage, rounding, and karyorrhexis. Cu B, cucurbitacin B; Doxa, doxazosin; CCK-8, counting kit-8; WPMY-1, human normal prostate stromal immortalized cell line; HPRF, human prostate fibroblasts. Scale bar = 50 µM.

The influence of Cu B on the proliferation and viability of the human prostate stromal immortalized cell line (WPMY-1) and human prostate fibroblasts (HPRF) was studied through the utilization of a CCK-8 assay. Cu B (12.5 nM–200 nM) inhibits the growth of WPMY-1 and HPRF cells in a dose- and time-dependent manner. WPMY-1 cells were more responsive to Cu B than HPRF cells. In WPMY-1 and HPRF cells, doxazosin (40 μM) resulted in a greater ability to inhibit prostate cell proliferation compared with Cu B (12.5 nM–200 nM) (Figure 2). After 48 h of Cu B treatment, the half-inhibition concentrations (IC50) of WPMY-1 and HPRF were 66.42 nM and 88.94 nM, respectively. After 72 h of treatment, the IC50 values of WPMY-1 and HPRF were 38.84 nM and 65.98 nM, respectively (Table 1). Moreover, cytomorphological observation has indicated that, compared with the control group, Cu B treatment induced distinct morphological alterations, manifesting with cell shrinkage, rounding, and karyorrhexis (Figure 3).

### 2.2. Cu B Induced the Apoptosis of WPMY-1 and HPRF Cells

**Figure 4:**
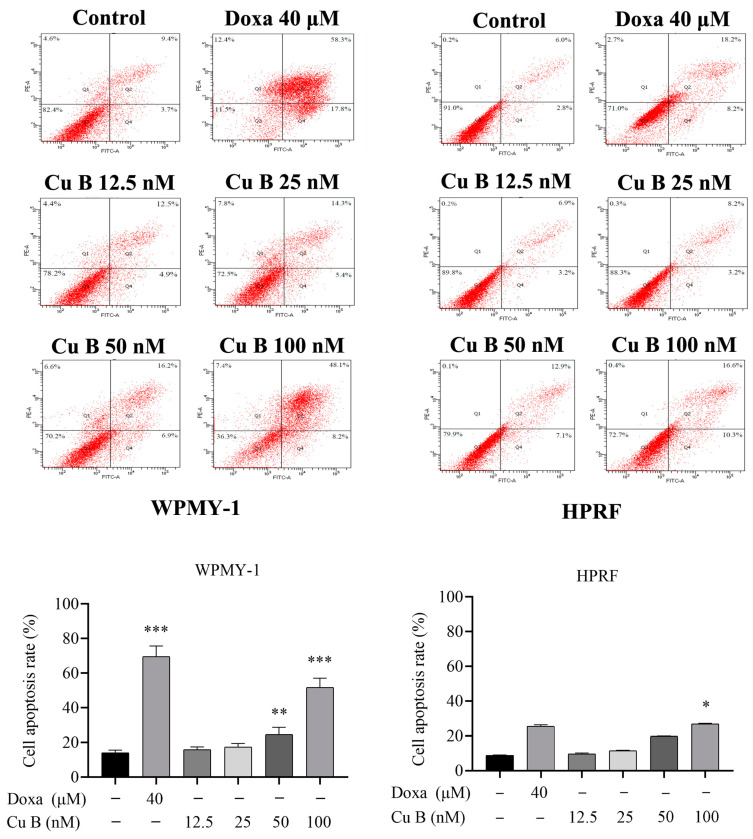
Apoptosis of WPMY-1 and HPRF that were treated with Cu B and Doxa. Cu B and Doxa induced the apoptosis of prostate cells based on the results of flow cytometry analysis. In WPMY-1 cells, the apoptosis rate of the Doxa group and the middle-to-high concentration Cu B (50 nM–100 nM) group was significantly increased compared with the control group. In HPRF cells, only the apoptosis rate of the high-concentration Cu B (50 nM–100 nM) group was significantly increased compared with the control group. The data are presented as mean ± SD of three independent experiments, analyzed using one-way ANOVA followed by least significant difference post-hoc test or Dunnett’s post-hoc test. * *p* < 0.05, ** *p* < 0.01, *** *p* < 0.001, compared with the control group. Cu B, cucurbitacin B; Doxa, doxazosin; WPMY-1, human normal prostate stromal immortalized cell line; HPRF, human prostate fibroblasts.

To further evaluate the efficacy of Cu B on prostate cells, we used flow cytometry to detect the apoptosis rate of cells after Cu B treatment. Annexin V-FITC/PI staining showed that after Cu B treatment, WPMY-1 and HPRF exhibited concentration-dependent apoptosis, and WPMY-1 was more sensitive than HPRF cells. Compared with the control group, prostate cells in the high-dose Cu B group (100 nM) showed significant apoptosis. In addition, 100 nM Cu B and 40 μM doxazosin had almost similar apoptosis-inducing abilities in HPRF cells (Figure 4).

### 2.3. Cu B Caused G2/M Cell Cycle Arrest in WPMY-1 Cells

**Figure 5:**
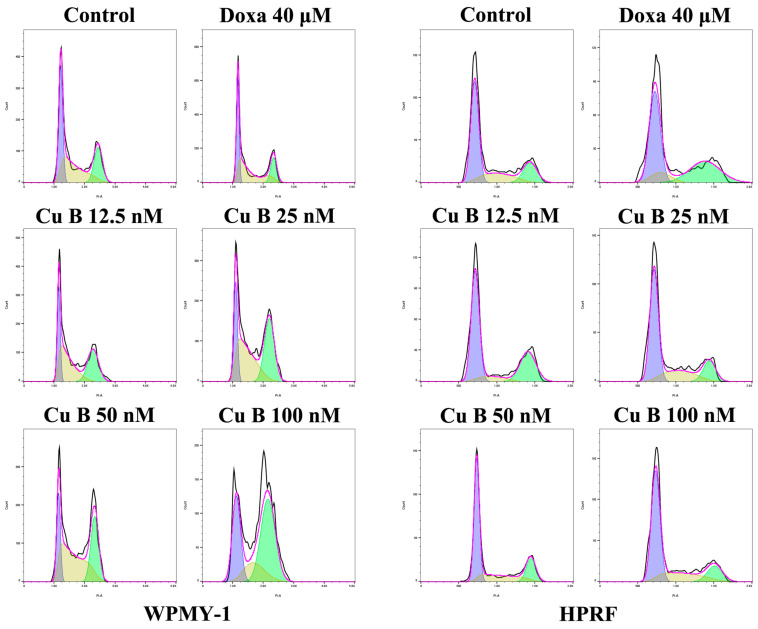

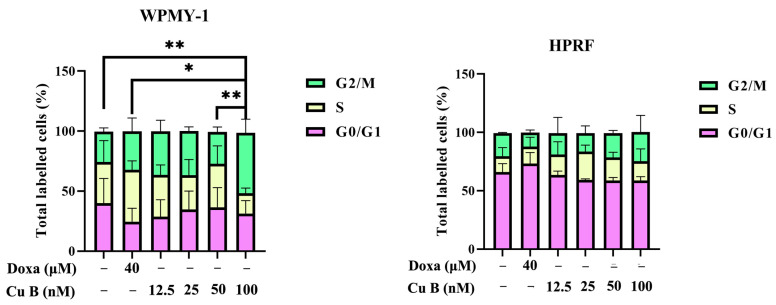
Cell cycle results of WPMY-1 and HPRF cells treated with Cu B and Doxa. Flow cytometry analysis showed that Cu B could block the G2/M phase of WPMY-1 cells. In WPMY-1 cells, the proportion of G2/M phase cells in the high-concentration Cu B (100 nM) group was significantly increased compared with the control group, Doxa group, and Cu B (50 nM) group. No significant changes were observed in the proportion of cells in HPRF cells. The data are presented as mean ± SD of three independent experiments, analyzed using one-way ANOVA followed by least significant difference post-hoc test or Dunnett’s post-hoc test. * *p* < 0.05, ** *p* < 0.01. Cu B, cucurbitacin B; Doxa, doxazosin; WPMY-1, human normal prostate stromal immortalized cell line; HPRF, human prostate fibroblasts.

To further investigate the effect of copper B on prostate cell proliferation, we used flow cytometry to detect the effect of copper B on the cell cycle of WPMY-1 cells and HPRF cells. PI staining showed that after Cu B treatment, the proportion of WPMY-1 cells in the G2/M phase in the Cu B (100 nM) group increased significantly compared with the control group and the Cu B (50 nM) group. However, Cu B treatment had no significant effect on the cell cycle of HPRF cells (Figure 5).

### 2.4. Cu B Affected p53 Signaling Pathway in WPMY-1 Cells

**Figure 6:**
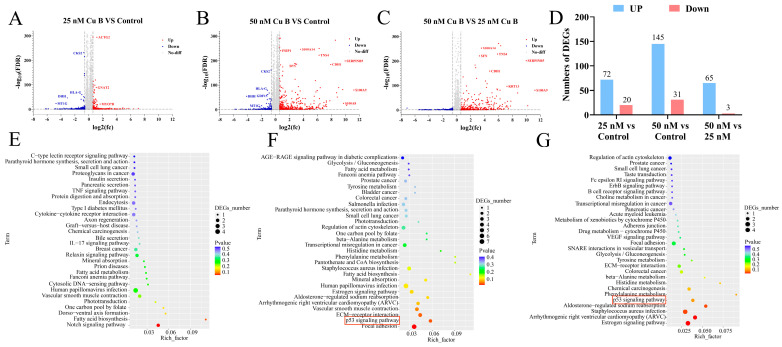
RNA-sequencing results of DEGs and KEGG pathway enrichment analysis in WPMY-1 cells after Cu B treatment for 48 h. Volcano chart (**A**–**C**) and column chart (**D**) showing the number of DEGs up- and down-regulated in the Cu B (25 nM) group versus the control, Cu B (50 nM) group versus the control, and Cu B (50 nM) group versus Cu B (25 nM) group. (**E**) KEGG enrichment analysis of DEGs between the Cu B (25 nM) group and the control group. (**F**) KEGG enrichment analysis of DEGs between the Cu B (50 nM) group and the control group (*p* = 0.037213701 for p53 signaling pathway). (**G**) KEGG enrichment analysis of DEGs between the Cu B (50 nM) group and the Cu B (25 nM) group. Cu B, cucurbitacin B; DEGs, differentially expressed genes; KEGG, Kyoto Encyclopedia of Genes and Genomes. (*n* = 3).

To study the underlying molecular mechanism of Cu B-induced WPMY-1 cell apoptosis, we performed RNA-sequencing (RNA-seq) to screen differentially expressed genes (DEGs) between the control and Cu B-treated groups. A total of 92 and 176 DEGs were obtained in Cu B (25 nM) and Cu B (50 nM)-treated WPMY-1 cells, respectively, compared with cells treated with 0.1% DMSO, which included 72 up-regulated and 20 down-regulated DEGs in Cu B (25 nM) group (Figure 6A,D) and 145 up-regulated and 31 down-regulated DEGs in Cu B (50 nM) group (Figure 6B,D). Moreover, compared with the Cu B (25 nM) group, sixty-eight DEGs were obtained in the Cu B (50 nM) group, involving sixty-five up-regulated and three down-regulated DEGs (Figure 6C,D). The functional analysis of all DEGs was carried out using the KEGG pathway enrichment analysis. Most DEGs were associated with signaling pathways, including p53, IL-17, Notch, VEGF, and prostate cancer pathways. Among them, the p53 signaling pathway was involved in the Cu B (50 nM) group versus the control group and Cu B (50 nM) group versus the Cu B (25 nM) group, and p53 signaling was one of the most significantly enriched pathways after Cu B (50 nM) treatment (Figure 6E–G).

### 2.5. Cu B Regulated Gene Expression Levels of p53/MDM2 Signaling Axis

**Figure 7:**
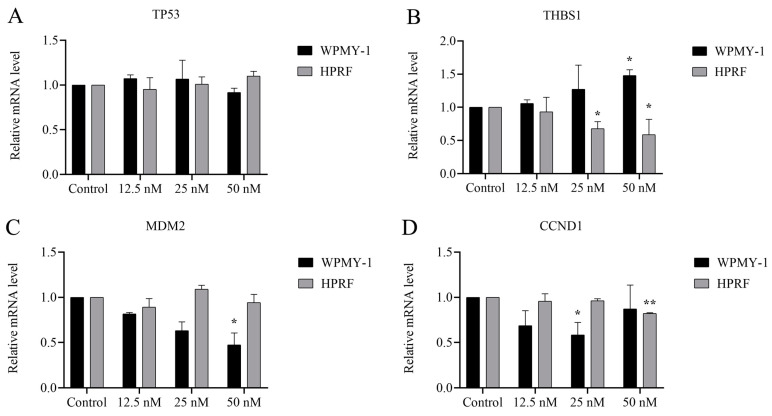
Cu B regulated the expression of selected genes in WPMY-1 and HPRF cells based on RT-qPCR results. (**A**) The gene expression level of *TP53* in Cu B-treated cells. (**B**) The gene expression level of *THBS1* in Cu B-treated cells. (**C**) The gene expression level of *MDM2* in Cu B-treated cells. (**D**) The gene expression level of *CCND1* in Cu B-treated cells. In WPMY-1 cells, compared with the control group, the expression of *MDM2* and *CCND1* genes was significantly down-regulated under the action of Cu B (50 nM) and Cu B (25 nM), respectively; the expression of *THBS1* gene was significantly up-regulated under the action of Cu B (50 nM). In HPRF cells, compared with the control group, the expression of *CCND1* gene was significantly up-regulated under the action of Cu B (50 nM); the expression of *THBS1* gene was significantly down-regulated under the action of Cu B (25 nM–50 nM). The expression of *TP53* gene was not affected by Cu B. The data are presented as mean ± SD of three independent experiments, analyzed using one-way ANOVA followed by least significant difference post-hoc test or Dunnett’s post-hoc test. * *p* < 0.05, ** *p* < 0.01, compared with control. Cu B, cucurbitacin B; TP53, tumor protein p53; THBS1, thrombospondin 1; MDM2, mouse double minute-2; CCND1, cyclin D1; WPMY-1, human normal prostate stromal immortalized cell line; HPRF, human prostate fibroblasts; RT-qPCR, real-time quantitative polymerase chain reaction.

RNA sequencing analysis demonstrated that Cu B (50 nM) treatment resulted in a marked enrichment of the p53 signaling pathway. To gain further insights, we utilized real-time quantitative polymerase chain reaction (RT-qPCR) to determine the relative mRNA levels of p53 and its downstream target gene. The results suggested that in WPMY-1 cells, p53-regulated *MDM2* and *CCND1* gene levels were significantly down-regulated in the high (50 nM) and medium (25 nM) Cu B groups (Figure 7C,D). The conspicuously up-regulated *THBS1* expression level was found in the high-dose Cu B group in WPMY-1 cells (Figure 7B). Simultaneously, Cu B (50 nM) down-regulated *THBS1* and *CCND1* expressions in HPRF cells (Figure 7B,D). Of note, Cu B had no significant effect on the *TP53* gene expression level (Figure 7A).

### 2.6. Cu B Up-Regulated TP53 and THBS1 Protein Expressions and Modulated MDM2 Protein Expression in Prostate Cell

**Figure 8:**
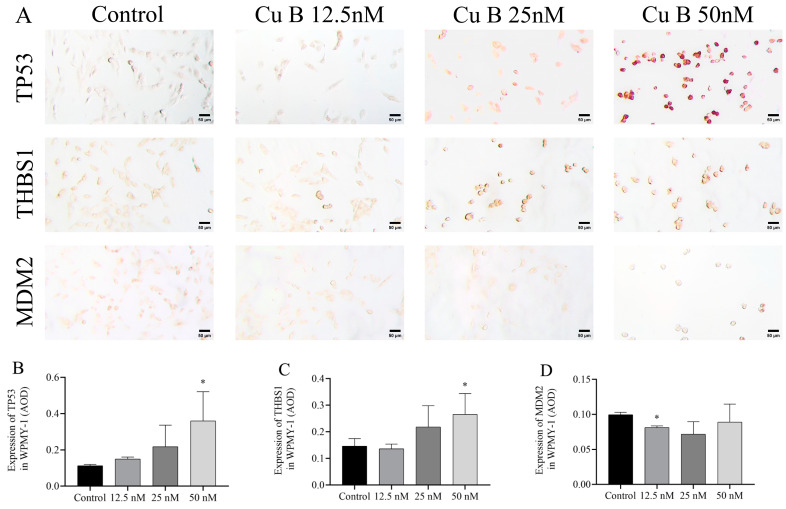
Impact of Cu B on the modulation of TP53, THBS1, and MDM2 protein expressions in WPMY-1 cells. (**A**) Immunocytochemistry images of TP53, THBS1, and MDM2 in WPMY-1 cells (400×). (**B**) Effect of Cu B on TP53 expression. (**C**) Effect of Cu B on THBS1 expression. (**D**) Effect of Cu B on MDM2 expression. Scale bar = 50 μm. In WPMY-1 cells, compared with the control group, the high-dose Cu B (50 nM) group significantly up-regulated the expression of TP53 and THBS1, while the low-dose Cu B (12.5 nM) group significantly down-regulated the expression of MDM2. The data are presented as mean ± SD of three independent experiments, analyzed using one-way ANOVA followed by least significant difference post-hoc test or Dunnett’s post-hoc test. * *p* < 0.05, compared with the control. Cu B, cucurbitacin B; TP53, tumor protein p53; THBS1, thrombospondin 1; MDM2, mouse double minute-2; AOD, average optical density.

**Figure 9:**
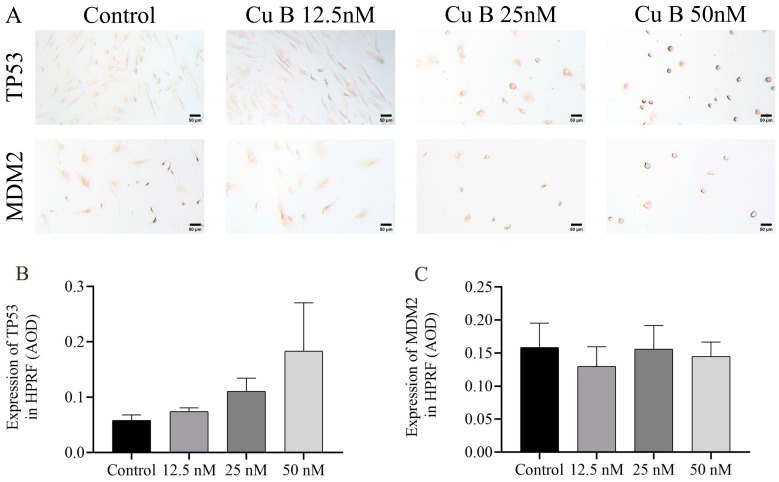
Impact of Cu B on the modulation of TP53 and MDM2 protein expressions in HPRF cells. (**A**) Immunocytochemistry images of TP53 and MDM2 in HPRF cells (400×). (**B**) Effect of Cu B on TP53 expression. (**C**) Effect of Cu B on MDM2 expression. Scale bar = 50 μm. Cu B had no significant effect on the expression of TP53 and MDM2. The data are presented as mean ± SD of three independent experiments, analyzed using one-way ANOVA followed by least significant difference post-hoc test or Dunnett’s post-hoc test. Cu B, cucurbitacin B; TP53, tumor protein p53; MDM2, mouse double minute-2; HPRF, human prostate fibroblasts; AOD, average optic density.

**Figure 10:**
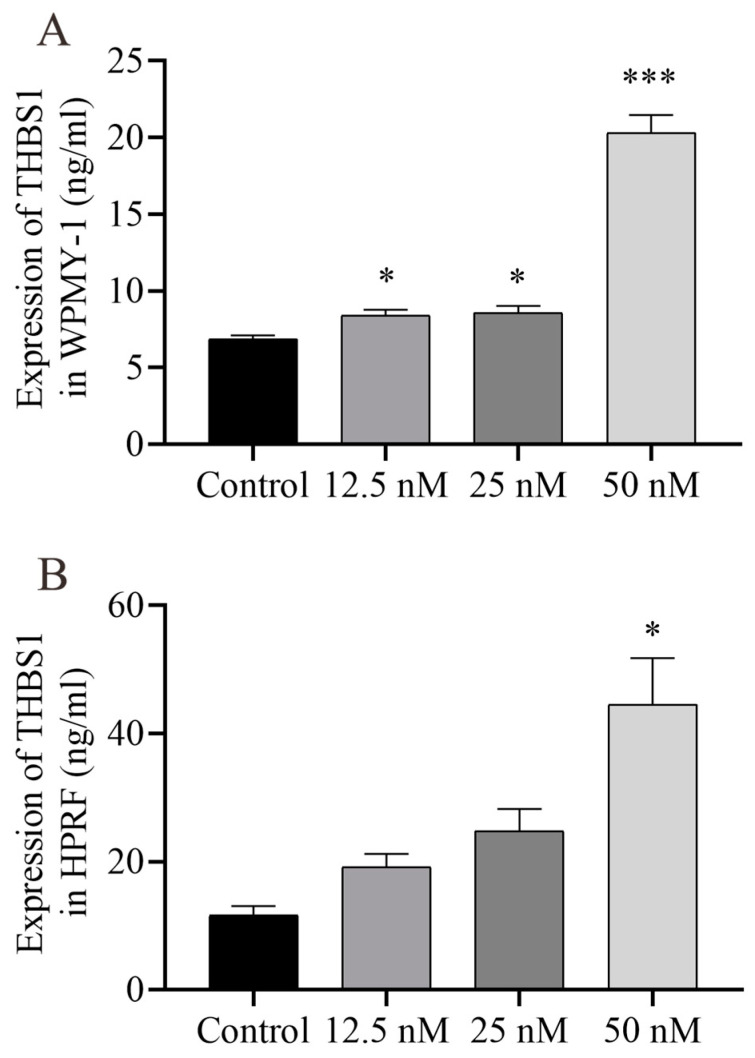
ELISA analysis results of THBS1 in the prostate cell culture supernatant. Prostate cells were treated with vehicle (0.1% DMSO) and Cu B (12.5 nM, 25 nM, 50 nM) for 48 h. (**A**) THBS1 level in WPMY-1 cells. (**B**) THBS1 level in HPRF cells.; WPMY-1: human normal prostate stromal immortalized cell line; HPRF: human prostate fibroblasts. Cu B significantly increased the expression of THBS1 in two prostate cell types. The data are presented as mean ± SD of three independent experiments, analyzed using one-way ANOVA followed by least significant difference post-hoc test or Dunnett’s post-hoc test. * *p* < 0.05, *** *p* < 0.001, compared with the control. ELISA, enzyme-linked immunosorbent assay; THBS1, thrombospondin 1; DMSO, dimethyl sulfoxide; Cu B, cucurbitacin B.

To conduct a more thorough and orderly study of the molecular mechanism of Cu B and the regulation of the p53 signaling pathway, immunocytochemistry techniques were employed to evaluate the protein expression levels related to p53. In WPMY-1 cells, the results of MDM2 and THBS1 protein expression were down-regulated and up-regulated compared with the control group, respectively, which was directly consistent with the RT-qPCR results; the high-dose Cu B group significantly induced the expression of TP53 (Figure 8A–D), which was inconsistent with the RT-qPCR results. In HPRF cells, Cu B had no significant effect on the expression of TP53 and MDM2 proteins (Figure 9). Considering that THBS1 is a secreted protein, we also used enzyme-linked immunosorbent assays (ELISA) to detect the secretion of THBS1 in the culture supernatants of two prostate cell lines. The results showed that Cu B significantly increased the secretion of THBS1 in WPMY-1 and HPRF cells (Figure 10).

## 3. Discussion

Doxazosin is a selective α1 adrenergic receptor blocker that promotes vasodilation and decreases peripheral vascular resistance by inhibiting the binding of norepinephrine to α1 receptors on the membrane of vascular smooth muscle cells. Clinically, this drug is often used to treat benign prostatic hyperplasia. In this study, by observing cell proliferation, we screened 40 μM doxazosin as a positive control, a concentration that can effectively compare the phenotypic differences between Cu B and doxazosin-treated prostate cells. From a clinical perspective, benign prostatic hyperplasia is principally contingent on prostatic stromal proliferation [19]. The ratio of stromal cells to epithelial cells reached 5:1 in benign prostatic hyperplasia tissues [20]. WPMY-1, a myofibroblast stromal cell line, was derived from SV40 large-T antigen-immortalized stromal cells and expressed smooth muscle α-actin and vimentin [21]. Human prostate fibroblasts (HPRF) are mesenchymal cells derived from embryonic mesoderm. The uncontrolled proliferation of stromal cells contributes to the pathological process of prostate lesions [22]. Thus, prostate fibroblasts and stromal cells have been extensively used in prostatic hyperplasia studies. For instance, resveratrol possesses the capacity to initiate G0/G1 phase cell cycle arrest and attenuate the activity of nuclear factor-κB, ultimately suppressing cell growth and prompting apoptosis in WPMY-1 cells [23]. Another case in point is that prolonged exposure to the 5α-reductase inhibitor leads to down-regulation of IGF-1 expression in HPRF, which triggers the activation of autophagic flow via the mTOR pathway and induces autophagy in prostate epithelial cells [24]. In this study, we used the WPMY-1 and HPRF cell lines to evaluate the anti-proliferative activity of Cu B in vitro. These results highlight the ability of Cu B to effectively inhibit prostate cell proliferation and induce apoptosis. We speculate that Cu B exerts an inhibitory effect on prostate cells, which may manifest as an up-regulation of p53 and THBS1 expression levels and a down-regulation of CCND1 and MDM2 expression levels in prostate cells. Therefore, we hypothesize that this inhibitory effect is achieved by Cu B regulating gene expression related to the p53/MDM2 axis.

The *TP53* gene, commonly referred to as the *p53* gene, serves as a crucial tumor suppressor, playing a pivotal role in preserving the integrity of cellular DNA. Mutations in the *p53* tumor suppressor gene occur in approximately half of all human cancers, highlighting its significance as a crucial tumor suppressor [25]. In normal cells, the expression of *p53* is typically low, whereas it is significantly up-regulated in malignant tumors [26]. Research has demonstrated that p53 plays a pivotal role in numerous biological functions, encompassing cell cycle arrest, apoptosis, and cellular differentiation [27]. Mechanistically, p53’s activity is modulated by its negative regulator, MDM2. The human *MDM2* gene resides on chromosome 12q 12.3-q15 and encodes a protein with 491 amino acid residues, which facilitate the formation of numerous functional binding sites [28]. *MDM2* is generally considered to be an oncogene, and different types of tumors, including breast, pancreatic, and bladder cancers, exhibit *MDM2* amplification [29,30,31]. The *MDM2* gene comprises both the P1 and P2 promoters, with the P2 promoter serving as a negative regulator of *p53*. MDM2 is an E3 ubiquitin ligase that can down-regulate p53 activity through three mechanisms: (1) targeting p53 for proteasome-mediated degradation by polyubiquitination; (2) exporting p53 from the nucleus by monoubiquitination; and (3) directly binding to p53, blocking its transcriptional activation of key targets [32]. To an extent, MDM2 overexpression is associated with a poor survival rate and chemotherapeutic resistance. MDM2 may play a pivotal supporting role in the prediction of disease outcomes and is emerging as a promising therapeutic target for the treatment of human malignancies [33,34]. In vivo studies demonstrated that the interaction between MDM2 and E-cadherin promoted breast cell motility and invasion by inducing the ubiquitination and degradation of E-cadherin [35]. In our study, Cu B down-regulated the *MDM2* mRNA level but did not affect the *TP53* transcriptional level in WPMY-1 cells. It is noteworthy that the addition of 50 nM Cu B significantly enhanced the expression of p53 protein in WPMY-1 cells, implying that Cu B does not directly induce *p53* transcriptional activation but likely contributes to *p53* translational or protein stability modulation. The aforementioned study may underlie the groundwork that Cu B inhibits prostatic cell proliferation.

However, contrary to the changes in *MDM2* gene and protein expression in WPMY-1 cells, our research group found in previous studies that BPH-1 cells exhibited up-regulated *MDM2* gene and protein expression after Cu B treatment [18]. The differences in these observations can be attributed to various factors, including differences in the sensitivity of prostate cells to Cu B, diversity in protein functions, genetic perturbations, and protein dynamics. In our study, Cu B inhibited the proliferation of prostate cells such as WPMY-1, HPRF, and BPH-1, but the sensitivity of these three prostate cells to Cu B varied. WPMY-1 cells were the most sensitive to Cu B, and BPH-1 cells were more sensitive to Cu B than HPRF cells. The differences in sensitivity among different cells suggest that higher concentrations of Cu B or longer administration times may induce changes in the expression of corresponding genes and proteins in HPRF cells. From the perspective of gene polymorphism, thousands of genes are differentially affected by copy number variations or differential expressions in different prostate cell lines. In this context, genes involved in drug transport and metabolism, drug target maintenance, cell survival, and cell death signaling present diverse genetic and transcriptional landscapes among different cell lines. In other words, differences in drug sensitivity can be attributed to various genetic factor perturbations, and differences in gene expression among different cells lead to differences in cellular sensitivity to drugs [36]. MDM2 is a multifunctional protein that has been reported to have a relatively high basal expression in BPH-1 cells. The partial or complete silencing of the *MDM2* gene in BPH-1 cells resulted in a marginal improvement in their migratory ability. However, the down-regulation of MDM2 protein expression in these cells diminished their responsiveness to docetaxel therapy [37]. Additionally, the MDM2 protein plays a crucial role in inhibiting mitotic progression and cell proliferation [38,39]. From the perspective of dynamic proteomics, although most proteins behave consistently in different cells, a small subset exhibits bimodal behavior, with a sharp increase in cellular variability approximately one day after drug administration. Upon exposure to drugs, intracellular target proteins undergo rapid translocation, manifesting as varying degrees of protein degradation or accumulation. Different cells can form subpopulations with distinct protein dynamics [40]. Therefore, human cells can be regarded as a dynamically regulated system, and due to differences in cellular sensitivity to drugs, protein functional diversity, genetic perturbations, and protein dynamics, gene and protein levels may exhibit different trends in different prostate cells. Based on the above research, we believe that MDM2 protein may have different regulatory functions in different types of prostate cells, which is one of the reasons for the phenomenon that Cu B induces the up-regulation of MDM2 protein expression in BPH-1 cells. However, whether MDM2 plays a key role in the inhibition of prostate cell proliferation by Cu B remains to be further investigated.

Since its identification as an angiogenesis inhibitor in 1990, THBS1 has attracted significant interest due to its role in tumor biology and potential as a therapeutic target. As a downstream target of P53, THBS1 regulates the secretion of various proteins, including TGF-β1, proteases, angiogenic growth factors, and secreted frizzled-related protein (sFRP)-1 [41]. The down-regulation of THBS1 expression in glioblastoma multiforme suggests a potential role for THBS1 promoter methylation and transcriptional silencing in this process [42]. In the tumor microenvironment, THBS1 has the potential to be utilized as a biomarker for interstitial colorectal cancer detection, and it plays a pivotal role in suppressing anti-tumor immunity [43]. THBS1 was found to be a direct target of epigenetic suppression by the enhancer of zeste homolog 2 (EZH2), which can directly regulate THBS1, thereby promoting neuroendocrine progression and angiogenesis in invasive prostate cancer [44]. Additionally, Histone deacetylase-2 (HDAC2) inhibited the expression of THBS1, leading to the stimulation of angiogenesis and the acceleration of prostate cancer development via beta-adrenergic signaling [45]. The evidence presented demonstrates that THBS1 serves as a promising therapeutic target for cancerous malignancies or proliferative vascular disorders. The above evidence suggests that THBS1 may be a promising therapeutic target for cancer, malignant tumors, or proliferative vascular diseases. In vitro studies have shown that allicin, a natural compound, has the ability to trigger apoptosis and cell cycle arrest in breast cancer cells, which is attributed to its regulation of protein expression related to p53 signaling, such as THBS1 [46]. In our study, we found that the regulatory trend of Cu B in WPMY-1 cells and HPRF cells was opposite at the genetic level, up-regulating the gene level of *THBS1* in WPMY-1 cells and down-regulating the gene level of *THBS1* in HPRF cells. At the protein level, considering that THBS1 is a secreted protein, we detected the culture supernatant levels of two prostate cell lines and found that the gene expression level of THBS1 increased in both cell lines. These results emphasize that Cu B significantly up-regulates the protein expression of THBS1, indicating that THBS1 may play a partial role in the apoptotic response after Cu B treatment. The difference in mRNA and protein expression of THBS1 in prostate cells may be due to the direct regulation of THBS1 protein expression by Cu B, and the Cu B-induced *THBS1* gene and its protein expression may form a negative feedback loop in HPRF cells, where the expression of THBS1 protein may inhibit the transcription of the *THBS1* gene and form an opposite trend. We need to conduct molecular mechanism studies to elucidate more about the complex process of p53 dependency, including the roles of MDM2 and THBS1 in inhibiting cell proliferation. Therefore, it would be very meaningful to further explore whether Cu B treatment induces functional interactions between p53, MDM2, and THBS1.

The *CCND1* gene includes five exons separated by four introns, which encode the full-length canonical cyclin D1 product of 295 amino acids [47]. CCND1 functions as a cell apoptosis and cycler regulator and modulates multiple physiological and pathological processes. Overexpression of CCND1 is often observed in a different spectrum of tumor types, including breast cancer [48], neck cancer [49], colorectal cancer [50], and prostate cancer [51]. Knockdown of *CCND1* inhibited glioblastoma cell proliferation and invasion by simultaneously targeting multidrug resistance protein 1 (MDR1), B-cell lymphoma-2 (Bcl-2), and caspase-3 [52]. A detailed molecular and biological mechanism investigation confirmed that the p53-inducible gene *PC3* arrests G1/S progression by inhibiting pRb function, ultimately down-regulating CCND1 expression and initiating antiproliferative activity [53]. Accumulating studies have documented that natural products, oleanolic acid and standardized cornus officinalis, could inhibit prostate cell proliferation by down-regulating the gene expressions of proliferating cell nuclear antigen (PCNA), CCND1, and cyclin E [54,55]. Our evidence showed that Cu B treatment significantly inhibited the p53 downstream target gene *CCND1*, and the down-modulation of CCND1 may promote Cu B-induced prostate cell apoptosis. However, the *p53* gene is also related to cell cycle arrest [56]. In addition, some studies have found that Cu B can induce cell cycle arrest [57,58]. In this experiment, we used flow cytometry technology to observe that WPMY-1 cells treated with Cu B would trigger periodic arrest in the G2/M phase, thereby inhibiting cell proliferation. However, similar periodic arrests were not observed in HPRF cells. Therefore, further research on this phenomenon and the exploration of its mechanisms will be the key direction of future work.

## 4. Materials and Methods

### 4.1. Chemicals

Cucurbitacin B (CAS No. 6199-67-3, 99.91% pure, liquid chromatography mass spectrophotometer; formula weight, 558.7) was derived from MedChemExpress (Shanghai, China). Doxazosin (CAS No. 74191-85-8, ≥98% pure, infrared spectrum; formula weight, 451.48) was purchased from Macklin Biochemical Technology Co., Ltd. (Shanghai, China). The Cell Counting Kit-8 (CCK-8), cell cycle detection kit, DAB horseradish peroxidase color development kit, enhanced immunostaining permeabilization buffer, 4% paraformaldehyde fix solution, QuickBlock™ blocking buffer for immunol staining, and QuickBlock™ primary and secondary antibody dilution buffer for immunohistochemistry were all purchased from Beyotime Biotechnology (Shanghai, China). Annexin V-FITC/PI kits were obtained from Yeasen (Shanghai, China).

### 4.2. Cell Culture

The human normal prostate stromal immortalized cell line (WPMY-1) and human prostate fibroblasts (HPRF) were purchased from Zhong Qiao Xin Zhou Biotechnology Co., Ltd. (Shanghai, China). WPMY-1 cells were incubated in Dulbecco’s Modified Eagle Medium (DMEM), supplemented with 5% fetal bovine serum (FBS) and 1% penicillin-streptomycin, to maintain their growth. Similarly, HPRF cells were grown in fibroblast medium (FM) enriched with 1% fibroblast growth supplement, 2% FBS, and 1% penicillin-streptomycin. Both cell lines were cultured in an incubator maintained at 37 °C with 5% CO_2_.

### 4.3. Cell Viability and Cell Morphology

Cells in the logarithmic growth phase were trypsinized to create a single-cell suspension and then counted using a hemocytometer. Prostate cells, either 4000 or 3000 cells per well, were seeded onto 96-well plates with six replicate wells and incubated for 24 h at 37 °C in a 5% CO_2_ environment. Subsequently, based on the results of the pre-experiment, the drug concentration gradient was set to double, and these cells were exposed to different treatments: vehicle (0.1% DMSO (*v*/*v*)), doxazosin (40 μM), or Cu B (12.5, 25, 50, 100, 200 nM) for durations of 48 h and 72 h. Afterward, 10 μL of CCK8 reagent was dispensed into each well and incubated for 1–4 h at 37 °C. The optical density of each well was then determined using a microplate reader set to 450 nm (BioTek, Winooski, VT, USA). The CCK-8 assays were replicated three times to ensure accuracy. The calculation of cell viability is determined using the following formula: Cell viability (%) = [(A_sample_ − A_blank_)/(A_vehicle_ − A_blank_)] × 100%. In this formula, A_sample_ represents the optical density of wells containing cells, CCK-8 reagent, and Cu B solution; A_blank_ corresponds to the optical density of wells with medium and CCK-8 reagent but without cells; A_vehicle_ denotes the optical density of wells that include cells and CCK-8 reagent but exclude Cu B solution.

### 4.4. Annexin V-FITC/PI Cell Apoptosis Detection

The analysis of cell apoptosis was enabled by the utilization of Annexin V-fluorescein isothiocyanate (FITC) and propidium iodide (PI) staining. WPMY-1 and HPRF cells were seeded in six-well plates at a lower density of 2.5 × 10^5^ cells per well for 24 h. Subsequently, the cells were exposed to different treatments: vehicle (0.1% DMSO (*v*/*v*)), doxazosin (40 μM), or Cu B (12.5, 25, 50, 100, 200 nM) for durations of 48 h. Following incubation, the cells underwent a single washing step with PBS, underwent trypsin digestion, and were subsequently centrifuged at 900 rpm for a period of 3 min. The supernatant of the cell suspension was discarded, and the cells were resuspended in 100 μL of binding buffer. Subsequently, 1 μL of Annexin V-FITC and 2 μL of PI staining solution were added to the resuspended cells. The apoptotic cells were incubated in the dark for 10–15 min at room temperature. After incubation, 400 μL of binding buffer was added to the samples and mixed thoroughly. The samples were then analyzed using flow cytometry (BD Biosciences, Franklin Lakes, NJ, USA) within 1 h of preparation. The data were analyzed using BD FACSDiva software v8.0.1 (Franklin Lakes, NJ, USA) to determine the apoptotic status of the cells.

### 4.5. Cell Cycle Assay

Cell cycle analysis was achieved through staining with propidium iodide (PI). WPMY-1 and HPRF cells were seeded into six-well plates at a density of 5.0 × 10^5^ cells per well and cultured for 24 h. Subsequently, the cells were subjected to different treatments: control (0.1% DMSO (*v*/*v*)), doxazosin (40 μM), or Cu B (12.5, 25, 50, 100, 200 nM) for a duration of 24 h. After completion of the incubation, the cells were washed once with PBS, digested with trypsin, and then centrifuged at 300× *g* for 5 min. The cell suspension was discarded, and the cells were resuspended in 2 mL of 75% ethanol for overnight fixation. The next day, the fixed cells were centrifuged at 300× *g* for 10 min. The cell suspension was discarded again, and the cells were resuspended in 1 mL of PBS and centrifuged at 300× *g* for another 10 min. The cell suspension was discarded once more, and the cells were vortexed and resuspended. To the resuspended cells, 500 μL of staining buffer, 25 μL of PI staining solution, and 10 μL of RNase A were added. The stained cells were incubated in the dark at 37 °C for 30 min. The samples were then analyzed using a flow cytometer (BD Biosciences, Franklin Lakes, NJ, USA) within 1 h after preparation. Data were analyzed using FlowJo software v10.8.1 (BD Biosciences, Franklin Lakes, NJ, USA) to determine the proportions of cells in each cell cycle phase.

### 4.6. RNA-Sequencing Analysis

Total RNA was isolated from the WPMY-1 cells using TRIzol^®^ Reagent according to the manufacturer’s instructions (Invitrogen, Shanghai, China), and genomic DNA was removed using DNase I (Takara, Shanghai, China). RNA quality was evaluated using 2100 Bioanalyser (Agilent, Shanghai, China), and the purity and concentration of RNA were determined using ND-2000 (NanoDrop Technologies, Shanghai, China). High-quality RNA sample (OD260/280 = 1.8~2.2, OD260/230 ≥ 2.0, RIN ≥ 6.5, 28S:18S ≥ 1.0, >10 μg) is used to construct sequencing library.

A total of 1 μg of total RNA was taken and prepared for RNA-seq transcriptome library using the TruSeqTM RNA Sample Preparation Kit from Illumina (San Diego, CA, USA). The main steps include the following: mRNA was isolated using oligo(dT) magnetic beads and fragmented using fragmentation buffer. Subsequently, the mRNA fragments underwent double-stranded cDNA synthesis, end repair, 3′ end A-base addition, and ligation of Illumina-indexed adaptors, following Illumina’s protocol. Then, the target cDNA fragments of 200–300 bp library size were selected on 2% Low Range Ultra Agarose and amplified using Phusion DNA polymerase (NEB) for 15 PCR cycles. After quantification by TBS380, paired-end libraries were sequenced using Illumina NovaSeq 6000 sequencing (150bp*2, Shanghai BIOZERON Co., Ltd., Shanghai, China). The sequencing data have been deposited in the NCBI Sequence Read Archive (SRA, http://www.ncbi.nlm.nih.gov/Traces/sra, accessed on 2 July 2024) under the accession number SRP517434.

Each group contained three biological replicates. The differentially expressed genes (DEGs) were selected with fold change > 1.5 and *p* value < 0.05 using R statistical package edgeR (Empirical Analysis of Digital Gene Expression in R, https://bioconductor.org/packages/release/bioc/html/edgeR.html, accessed on 23 August 2022). Gene Ontology (GO) functional enrichment and Kyoto Encyclopedia of Genes and Genomes (KEGG) pathway analysis were carried out using Goatools (https://github.com/tanghaibao/Goatools, accessed on 23 August 2022) and KOBAS (http://kobas.cbi.pku.edu.cn/home.do, accessed on 23 August 2022). DEGs were significantly enriched in GO terms and metabolic pathways when their Bonferroni-corrected *p*-value was less than 0.05.

### 4.7. Real-Time Quantitative Polymerase Chain Reaction (RT-qPCR)

Quantitative assessment of messenger RNA (mRNA) levels in prostate cells (*n* = 3) was conducted via real-time quantitative polymerase chain reaction (RT-qPCR) to compare the control group with Cu B-treated groups (12.5 nM, 25 nM, 50 nM). Efficient isolation of total RNA from prostate cells was achieved using the RNAeasyTM kit (R0026, Beyotime Biotechnology, Shanghai, China). RNA purity and concentration were determined using a spectrophotometer (ACT gene, Shanghai, China). cDNA synthesis was carried out by reverse transcription of 1 μg RNA on a PCR machine (Applied Biosystems, Shanghai, China), following the PrimeScript™RT Master Mix instructions (Takara, Shanghai, China). Primer sequences were designed using Premier Primer 6 and are detailed in Table 2. mRNA expression levels were determined using TB Green^®^Fast qPCR Mix (Takara, Shanghai, China) on a Roche LC480 instrument (Roche, Basel, Switzerland). Relative gene expression analysis was performed using the 2^−∆∆Ct^ method.

### 4.8. Immunocytochemistry

The WPMY-1 and HPRF cells were plated onto BD Biosciences cell culture slides (354104, Franklin Lakes, NJ, USA) at a concentration of 8000 cells per well. After an overnight incubation to promote cell attachment, the cells were treated with either a control solution (0.1% DMSO) or various concentrations of Cu B (12.5 nM, 25 nM, and 50 nM) for 24 h. Subsequently, the slides were rinsed three times with phosphate-buffered saline (PBS) to eliminate residual reagents, followed by fixation using 4% paraformaldehyde. Then, the slides were permeabilized with an immunostaining permeabilization buffer and blocked with QuickBlock™ blocking buffer for 20 min at room temperature to prevent nonspecific antibody binding. Cells were incubated overnight at 4 °C with the following primary antibodies: TP53 (1:100, AF0255, Beyotime, Shanghai, China), THBS1 (1:75, D162423, Sangon Biotech, Shanghai, China), and MDM2 (1:100, D160611, Sangon Biotech, Shanghai, China). Negative controls were created by excluding the primary antibodies from the procedure. Subsequently, the cells were exposed to either HRP-conjugated goat anti-mouse IgG (1:200, D110087, Sangon Biotech, Shanghai, China) or HRP-conjugated goat anti-rabbit IgG (1:200, D110058, Sangon Biotech, Shanghai, China) for 1 h at ambient temperature. Following this incubation, the cells were thoroughly washed three times with PBS to remove unbound antibodies. The staining process was then initiated using DAB staining solution. Afterward, the samples were carefully inspected and evaluated under an inverted microscope (AOSVI, Shenzhen, China). For each experimental group, three distinct cell sections were analyzed, and from each section, three random microscopic fields were chosen for further analysis. Using Image J software v1.8.0, the obtained cell section images are converted into grayscale images, and the gray values of the grayscale images are transformed into OD values. Then, the OD values of each point on the image are accumulated to obtain the Integrated Option Density (IOD) value, which is proportional to the total amount of the target protein. The IOD value is divided by the area of the target protein distribution region (IntDen/Area) to obtain the average density (average optical density, AOD), which reflects the concentration of the target protein per unit area. The semi-quantitative expression of TP53, THBS1, and MDM2 is detected through the AOD value.

### 4.9. Enzyme-Linked Immunosorbent Assay (ELISA)

WPMY-1 cells (4 × 10^5^ cells/well) and HPRF cells (3 × 10^5^ cells/well) were seeded in 6-well plates and incubated for 24 h. Following this, the cells were treated with Cu B (12.5 nM, 25 nM, 50 nM) for a duration of 48 h. Subsequently, the supernatants from the WPMY-1 and HPRF cells were collected by centrifugation. The concentration of THBS1 was determined using human ELISA Kits specific for THBS1 (JL11889, Jianglaibio, Shanghai, China) according to the manufacturer’s instructions. The protein levels of THBS1 in the WPMY-1 and HPRF cells were quantitatively analyzed using an enzyme-linked immunosorbent assay and measured at a wavelength of 450 nm using a spectrophotometer (BioTek, Vermont, VT, USA).

### 4.10. Statistical Analysis

The experimental results are expressed as the mean ± standard deviation (SD) of three biological replicates, and statistical analysis was performed using SPSS 26.0 software. After satisfying the normality test, one-way ANOVA was used for comparison between groups. When the variance homogeneity was met, the least significant difference (LSD) post-hoc test was used; otherwise, Dunnett’s post-hoc test was employed. Differences were considered statistically significant at a *p* value of <0.05. Visual representations of the data were created using GraphPad Prism 8.0 software (GraphPad Software, San Diego, CA, USA).

## 5. Conclusions

In summary, this study uncovered that Cu B potentially exerts a potent inhibitory effect on the proliferation of prostate cells, such as WPMY-1 and BPH-1, via the p53/MDM2 axis. Molecular mechanistic investigations have demonstrated that Cu B markedly elevates the expression levels of TP53 and THBS1 proteins within prostate cells while concurrently diminishing the expression of cyclin D1. These findings underscore the potential of Cu B as a promising therapeutic candidate for anti-prostatic hyperplasia, offering a robust molecular rationale. Nevertheless, it is imperative to conduct further studies to unravel potential cross-talk between p53 and other signaling cascades in Cu B-mediated apoptosis. Additionally, the lack of notable cell cycle arrest in HPRF cells induced by Cu B necessitates thorough exploration. As our research progresses, we will also delve into the in vivo efficacy of Cu B in mitigating BPH.

## Figures and Tables

**Table 1:** The IC50 value of Cu B’s inhibitory effect on prostate cells.

Cell Types	IC50 (48 h)	IC50 (72 h)
WPMY-1	66.42 nM	38.84 nM
HPRF	88.94 nM	65.98 nM

**Table 2:** Prime sequences for RT-qPCR.

Genes	Forward Primer (5′-3′)	Reverse Primer (5′-3′)
*TP53*	CACTAAGCGAGCACTGCCCAACA	GCCTCATTCAGCTCTCGGAACATCT
*MDM2*	TTGGCGTGCCAAGCTTCTCTGTG	ACCTGAGTCCGATGATTCCTGCTGA
*THBS1*	ATGGAGAATGCTGTCCTCGCTGTTG	CGGTTGTTGAGGCTATCGCAGGAG
*CCND1*	CGCCCTCGGTGTCCTACTTCAAATG	AGACCTCCTCCTCGCACTTCTGTTC
*GAPDH*	CAGGAGGCATTGCTGATGAT	GAAGGCTGGGGCTCATTT

## Data Availability

The data provided in this study are available upon request to the corresponding author.
